# Protective Effect of Crocin against Mitochondrial Damage and Memory Deficit Induced by Beta-amyloid in the Hippocampus of Rats

**DOI:** 10.22037/ijpr.2020.112206.13604

**Published:** 2021

**Authors:** Bahareh Sadat Yousefsani, Soghra Mehri, Jalal Pourahmad, Hossein Hosseinzadeh

**Affiliations:** a *Research Institute for Islamic and Complementary Medicine, Iran University of Medical Sciences, Tehran, Iran. *; b *Department of Traditional Pharmacy, School of Persian Medicine, Iran University of Medical Sciences, Tehran, Iran. *; c *Pharmaceutical Research Center, Pharmaceutical Technology Institute, Mashhad University of Medical Sciences, Mashhad, Iran. *; d *Department of Pharmacodynamics and Toxicology, School of Pharmacy, Mashhad University of Medical Sciences, Mashhad, Iran.*; e *Department of Pharmacology and Toxicology, Faculty of Pharmacy, Shahid Beheshti University of Medical Sciences, Tehran, Iran.*

**Keywords:** Crocin, Beta-amyloid, Alzheimer’s disease, Neuroprotective, Isolated mitochondria

## Abstract

Alzheimer’s disease is the most common form of dementia among the elderly. This progressive neurodegenerative disorder affects brain regions that control cognition, memory, language, speech, and awareness. As a potent antioxidant, crocin has been proposed to effectively manage the neurodegenerative disease. In this study, the recovery effects of crocin on the memory deficits caused by the intra-hippocampal injection of amyloid beta1-42 (Aβ1-42) were evaluated in rats. We also considered the protective effects of crocin on the mitochondrial damage caused by Aβ1-42. We examined the memory deficits of rats with the help of the Morris water maze. Then, we determined different mitochondrial toxicity endpoints caused by Aβ1-42, including mitochondrial ROS formation, lipid peroxidation, mitochondrial membrane potential collapse, mitochondrial outer membrane integrity, and cytochrome c release. Our results demonstrated that the behavioral signs of memory deficiency caused by Aβ1-42 significantly (*P < *0.01) reduced by both pretreatment and post-treatment with crocin (30 mg/kg). Furthermore, crocin prevented all the Aβ1-42 induced above referenced mitochondrial upstream toxic events leading to neuronal apoptosis. These results demonstrated that crocin is a promising preventive candidate for the potential treatment of Alzheimer’s disease. Furthermore, it seems that the antioxidant and neuroprotective effects of crocin are better seen when the compound is pretreated beforehand rather than introduced afterward in Aβ1-42 exposed mitochondria.

## Introduction

One of the most important problematic neurodegenerative diseases is Alzheimer’s disease (AD). AD is characterized by abnormal structures in the brain, such as intracellular tau neurofibrillary tangles and extracellular beta-amyloid plaques (1). AD is an epidemic type of dementia that affects 10% of people in their 60s, 20% in their 70s, and 30% in their 80s in the U.S (2). According to the severity of AD, the annual cost of disease surveillance worldwide exceeds $200 billion. Because the population is growing older, so we have more patients that suffer from AD. It is expected that in 2050, the number of these patients will double (3). There are several risk factors associated with the beginning and progression of AD, including age, smoking, low social engagement, head injuries, working conditions, and genetics (4). For example, research projects demonstrated that welding fumes lead to AD progression, and genetics has an effective role in AD (5).

Due to the sensitivity of Alzheimer’s topic and the health and social problems that this progressive neurodegenerative disorder causes, many studies have been carried out, especially its prevention and control methods. Although many studies have shown that abundant chemical and natural compounds can partially protect neurons, but further studies are needed, especially for mechanical evaluations (6-8).

The mitochondrion is one of the most important organelles that play an exclusive role in neuronal cell survival or death. Mitochondria supply energy for metabolism in the neurons. They are responsible for more than 90% of the cellular ATP generation. Mitochondria also regulate cellular death pathways (9, 10). Many studies and documents accepted the key role of mitochondrial dysfunction and oxidative stress damages in the pathogenesis of Alzheimer’s disease. Some papers demonstrated that mitochondrial malformations such as increased oxygen species generation and defective mitochondrial dynamic balance might be an event in the initiation of AD. Furthermore, mitochondrial injury leads to ATP decrease, which may lead to acidosis and different kinds of brain injury such as AD. Therefore, mitochondrial protection can be very effective in preventing AD (11-13).

Crocin has many pharmacological activities on different body organs, including the cardiovascular system, reproductive system, and nervous system. Crocin is a potent antioxidant that demonstrated protective effects on the nervous system, such as antidepressant effects, anticonvulsant effects, anxiolytic activity and learning and memory-enhancement (14-22). 

As it is clear, memory and learning impairments are the result of neurodegenerative disorders. Previous studies demonstrated that crocin by inhibiting the suppression of long-term potentiation in hippocampal neurons can protect them (23). It was also able to enhance memory function in the aging model through antiglycative and antioxidative properties, which end in suppressing brain inflammatory mediators (24). Rashedinia *et al.* evaluated the protective effect of crocin on acrolein-induced tau phosphorylation in the rat brain and they concluded the reduction of tau phosphorylation and Aβ1-24 concentration via modulation of mitogen-activated protein kinases (MAPKs) that expression is probably involved in the neuroprotective mechanism of crocin (25). Previous papers revealed that crocin has a potent radical scavenging effect. Therefore, the neuroprotective, anti-aging, anti-inflammatory, and antitumor activities of crocin may be due to its antioxidant effect (26). 

In this study, we wanted to know whether crocin, as an antioxidant and neuroprotective agent, could prevent or treat Alzheimer’s disease caused by Aβ1-42 by protecting the mitochondria, and we also want to find out the exact mitochondrial mechanisms effect of crocin. In addition, there are many reports on the preventive effects of crocin that we wanted to examine whether there is a difference between crocin preventative and therapeutic effects.

## Experimental

*Chemicals*

Aβ1-42, 4-2-hydroxyethyl-1-piperazineethanesulfonic acid (*HEPES*), rotenone (*Rot*), dimethyl sulfoxide (*DMSO*), D-mannitol, thiobarbutiric-acid (*TBA*), 2′,7′-Dichlorofluorescin diacetate (*DCFH-DA*), Tris–HCl, sodium-succinate, phosphate-buffered saline (*PBS*), sucrose, KCl, Na_2_HPO_4_, MgCl_2_, Rhodamine123 (*Rh123*), Coomassieblue, ethylene glycol bis (2-aminoethyl ether)-N, N, N0, N0-tetraacetic acid (*EGTA*), and xylazine and ketamine were purchased from Sigma Chemical Co. (St. Louis, MO, USA). All chemicals used for this experiment were of the best analytical and pharmaceutical grade available. Crocin was quantified through the original method in an aqueous saffron extract (27). Crocin was 95% pure. Aβ peptide1-42 was dissolved in PBS at a concentration of 20 µg/µL, aliquoted and stored at -80 ˚C until use (40).

*Animals*

Male rats of the Wistar strain weighing 220–250 g were obtained from the Faculty of Pharmacy, Shahid Beheshti University of Medical Sciences. All animal groups were housed in cages until tests and handled daily with free access to food and water and 12 h/12 light/dark cycle. The humidity and temperature were under control. The Shahid Beheshti and Mashhad University of Medical Sciences Animal Ethics Committee approved all animal manipulations.

*Surgery and microinjection*

Rat anesthetization was done using intraperitoneal combination injection of 100 mg/kg ketamine hydrochloride and 20 mg/kg xylazine. Rats also received penicillin (1.5 × 10^5^ U/rat) before placement in a stereotaxic device (Stoelting, USA). The surgical area was cleaned and dried. A drop of dental hemo stop was employed to decrease bleeding. Aβ1-42 was intra-hippocampally (IH) injected. For intra-hippocampal injection, stainless steel guide cannula was placed at the CA1 area of the hippocampus. Stereotaxic coordinates for the dorsal hippocampus were taken from the atlas of Paxinus and Watson (anterior-posterior, 3.8 mm; lateral, ±2.2 mm from the central line, and ventral, 2.7 down from the top of the skull) (28). Beta-amyloid or PBS (0.5 µL per site = 10 µg) was infused (0.5 mL/min). A Gas Tight Hamilton syringe with Teflon plunger stop (250 μL) was used for the microinjection technique and after each injection, the needle was left in the tissue for 2 min. Penicillin was administered daily, and the rats were allowed 21 days to recover from surgery and after that, behavioral testing (Morris water maze) was performed. 

*Experimental design*

**Scheme 1:**
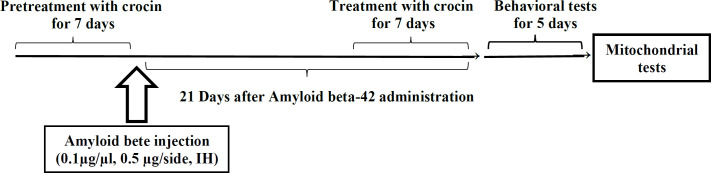
Schematic diagram of the injection and timeline of behavior and mitochondrial studies of all groups

In the present study, the animals were divided into the following groups (n = 7 for each group): 1) control group: had no surgical or dietary intervention. 2) Sham group: injected with the same volume of PBS (0.5 μL per side, IH). 3) Pretreatment with crocin group: received crocin (30 mg/kg, IP) daily in seven days before Aβ1-42 administration (0.1 µg/µL, 0.5 μL per side, IH). 4) Post-treatment with crocin group: received crocin (30 mg/kg, IP) daily in seven days after Aβ1-42 administration (0.1 µg/µL, 0.5 μL per side, IH). 5) Crocin group: only received crocin (30 mg/kg, IP) daily in seven days. 6) Aβ1-42 group: Just received Aβ1-42 (0.1 µg/mL, 0.5 μL per side, IH). Crocin dosage was selected based on the most effective dose of previous studies (24) (Scheme 1).

*Appraisement of spatial learning and memory by Morris water maze (MWM)*

The behavioral tests were performed 21 days after Aβ1-42 injection to assess spatial learning and memory of all the experimental groups. This maze included a black circular pool with a diameter of about 136 cm and a depth of 40 cm. The pool was filled with water to a depth of 35 cm (25 ± 2 °C). This black circular pool was divided into four equal quadrants. The target quadrant (north-west) has an invisible platform in its center that was 10 cm in diameter, which is located 1 cm under the water surface. Rat training was done for 4 days (one block consisting of four trials). In each trial, the rats were randomly placed in one of the four quadrants. A video camera was located just above the pool, linked to a computer and equipped with Ethovision software (Noldus Information Technology, Wageningen, Netherlands) that recorded the swimming pathway and related data. Animals were allowed to swim in the pool for a maximum of 90 s. If the rats did not find the hidden platform within this period, the researcher manually guided the animal to the platform. The rats were then rested on the platform for 20 s. Learning capabilities were measured by quantitative computer data in terms of escape latency (time to find the platform), traveled distance (path length to reach the platform), and swimming speed. After four training days, the rats were tested for probe trials. In the probe trial test, the platform in the target quadrant was removed. The rats were released on the opposite side of the target quadrant and freely swim for 90 s. The time each animal spent in the target quadrant was measured in the probe test (29).

*Evaluation of hippocampal mitochondrial function*

*Hippocampal mitochondrial isolation*

A day after completing MWM, all animals were sacrificed by cervical decapitation, and the hippocampal brain tissues were obtained. Then, with a glass handheld homogenizer, these tissues were minced and homogenized. Mitochondria were prepared from the rats’ hippocampi by differential centrifugation method (30). First, the samples were centrifuged (1500 g, 10 min, 4 °C), and the broken cell debris and nuclei were sedimented. The supernatant was exposed to centrifugation again (10000 g, 10 min, 4 °C). The upper layer was discarded, and the mitochondrial pellet was washed and suspended in the isolation medium and centrifuged again (10000 ×g, 10 min, 4 °C). Final suspensions of mitochondrial pellets were prepared in Tris buffer containing (0.05 M Tris–HCl, 0.25 M sucrose, 2.0 mMMgCl_2_, 20 mMKCl and 1.0 mMNa_2_HPO_4_, pH 7.4, 4 °C), except for mitochondrial samples used to measure the ROS level, mitochondrial membrane potential (MMP) and swelling, which were incubated in respiration buffer (10 mM Tris, 0.32 mM sucrose, 20 mM Mops, 0.5 mM MgCl_2_, 50 mM EGTA, 5 mM sodium succinate and 0.1 mM KH_2_PO_4_), MMP assay buffer (68 mM D-mannitol, 220 mM sucrose, 10 mMKCl, 5 mMKH_2_PO_4_, 2 mM MgCl_2_, 5 mM sodium succinate, 50 mM EGTA, 10 mM HEPES and 2 mM rotenone) and swelling buffer (3 mM HEPES, 70 mM sucrose, 230 mM mannitol, 5 mM succinate, 2 mMTris-phosphate and 1 mM of rotenone), respectively. Protein concentrations were measured using the Coomassie-blue protein-binding protocol as explained by Bradford (31). For the normalization process in all the following assays, the mitochondrial samples (0.5 mg mitochondrial protein per ml) were used. All the steps were operated on ice to guarantee high-quality mitochondrial preparation..

*Measurement of the hippocampal mitochondrial ROS levels*

The measurements of the mitochondrial ROS levels were performed by a fluorescence spectrophotometer using DCFH-DA. Briefly, isolated mitochondria were incubated with a respiration buffer (32), and DCFH-DA was added (final concentration, 10 mM) to the mitochondrial samples and then incubated for 10 min. After entry, DCFH-DA was hydrolyzed to non-fluorescent dichlorofluorescein (DCFH), which reacted with ROS and made highly fluorescent dichlorofluorescein (DCF). Then, the fluorescence intensity of DCF was quantified at 60 min using a Shimadzu RF5000U fluorescence spectrophotometer device at excitation and emission wavelengths of 500 nm and 520 nm, respectively (33).

*Measurement of the hippocampal MMP*

For the estimation of the MMP, the mitochondrial uptake of a cationic fluorescent dye, Rhodamine 123 (Rh123), was used. Mitochondrial fractions in the MMP assay buffer were incubated with 10 mM of Rh123. Then, the fluorescence was monitored at 60 min using the Shimadzu RF5000U fluorescence spectrophotometer device at an excitation wavelength of 490 and an emission wavelength of 535 nm (34).

*Measurement of hippocampal mitochondrial swelling*

The determination of hippocampal mitochondrial swelling was done through changes in light scattering measured spectrophotometrically at 540 nm (30 °C) (35). The isolated brain mitochondria were suspended in swelling buffer, and the absorbance was determined at 540 nm at 60 min with an ELISA reader apparatus (Tecan, Rainbow Thermo, and Austria). A reduction in absorbance was considered as an indicator of mitochondrial swelling.

*Measurement of cytochrome c oxidase activity and assessment of outer mitochondrial membrane damage*

Both mitochondrial outer membrane integrity and cytochrome c oxidase activity were measured using a cytochrome c oxidase assay kit (Sigma, St. Louis, MO). In the colorimetric trial, a decrease in absorbance was caused by oxidation of ferrocytochrome c at 550 nm to ferricytochrome c by cytochrome c oxidase. Experimental procedures were done according to the manufacturer’s protocol; 20 mg of isolated mitochondrial fraction were used for each reaction, and duplicate reactions were performed for each assay. For the determination of total mitochondrial cytochrome c oxidase activity, hippocampal mitochondrial fractions were diluted in the enzyme dilution buffer (10 mMTris–HCl, pH 7.0, containing 250 mM sucrose) with 1 mM n-dodecyl b-ᴅ-maltoside and placed on ice for 30 min. The reaction was performed by adding a fresh ferrocytochrome c substrate solution (0.22 mM) to the sample. The decrease in absorbance at 550 nm is linked to the oxidation of ferrocytochrome c by cytochrome-c oxidase. Cytochrome c oxidase activities were measured and normalized for protein per reaction, and the results were shown as units per milligram of mitochondrial protein. The mitochondrial outer membrane integrity was determined by evaluating the cytochrome-c oxidase activity of the mitochondria in the presence or absence of n-dodecyl b-ᴅ-maltosideas a detergent. The mitochondrial outer membrane damage was measured through the ratio between cytochrome-c oxidase activity eboth in the presence of detergent and its absence (35).

*Measurement of mitochondrial lipid peroxidation*

The MDA content was measured using the method of Zhang *et al.* (36). The mitochondrial fractions were incubated (1 h) with various concentrations of uranyl acetate at 30 °C; afterward, 0.25 mL sulfuric acid (0.05 M) was added to 0.2 mL mitochondrial fractions, with the addition of 0.3 mL TBA 0.2%. All the microtubes were put in a bath of boiling water for 30 min. Finally, the tubes were placed in an ice bath, and 0.4 mL n-butanol was added to each of them. Then, they were centrifuged (3500 g, 10 min). The amount of MDA formed in each sample was evaluated by measuring the absorbance of the supernatant at 532 nm with an ELISA reader apparatus (Tecan, Rainbow Thermo, Austria). Standard 1,1,3,3-Tetramethoxypropane (TEP) was used, and the MDA content was represented as µg/mg protein (36).

*Measurement of mitochondrial Glutathione (GSH) contents*

Mitochondrial GSH contents were evaluated using the spectrophotometer method and DTNB as the indicator for the isolated hippocampal mitochondria. The mitochondrial fractions were incubated with various concentrations of uranyl acetate (1 h) at 30 °C. Then the mitochondrial fractions (0.1 mL) were added into phosphate buffers (0.1 mol/L) and DTNB (0.04%) in a total volume of 3.0 mL (pH 7.4). At 412 nm, the yellow color was read on a spectrophotometer (UV-1601 PC, Shimadzu, Japan). The GSH amount was illustrated as µg/mg protein (37).

*Measurement of mitochondrial Cytochrome c Release*

The concentration of cytochrome-c was determined using the QAsntikine Rat/Mouse Cytochrome-c Immunoassay kit provided by R & D Systems, Inc. (Minneapolis, Minn) and the optical density of each well was determined using a spectrophotometer set to 450 nm (38).

*Statistical analysis*

The results for each group are presented as mean ± SD. GraphPad Prism-6 (GraphPad Software, La Jolla, CA) was used for the statistical analysis. A mean value for each dependent parameter of memory performance (traveled distance and escape latency) was evaluated over four trials in four training days. Mean values for each dependent measure of mitochondrial function were also calculated just a one-time point (1 h).

Statistical significance between the groups was determined by one-way analysis of variance (ANOVA) using a Bonferroni post hoc multiple comparison test. The *P*-value was set lower than 0.05.

## Results

*MWM results*

**Figure 1:**
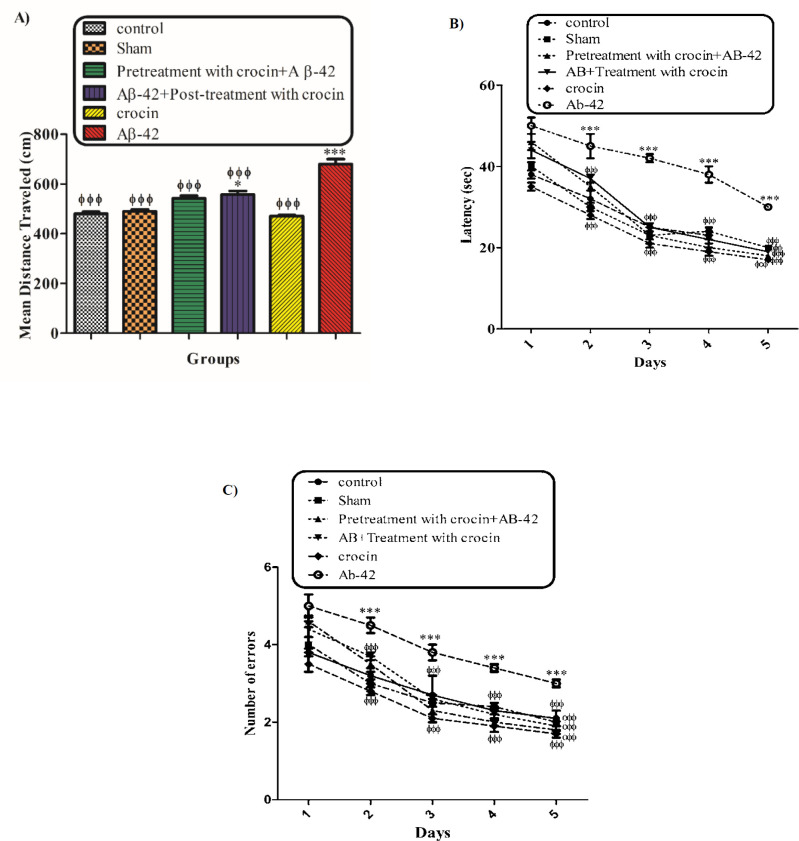
The protective effect of crocin (30 mg/kg) on Aβ1-42 (0.5 μL per side, IH) injected animals on the means of traveled distance (A) and escape latency (B) over 4 days in the MWM tests. ^*^P < 0.05, ^***^P < 0.001 compared to the control group. ^ϕϕ^ *P < *0.01, ^ϕϕϕ^ *P < *0.001 compare to the Aβ1-42 injected animals. The results for each group are presented as mean ± SD for 7 animals in each group. A mean value for each dependent parameter of memory performance was evaluated over four trials in four training days. Statistical significance between the groups was determined by one-way analysis of variance (ANOVA) using a Bonferroni post hoc multiple comparison test

The behavioral tests (MWM) were performed to evaluate the protective effect of crocin on Aβ1-42 induced memory deficiency in rats. Figure 1 shows the traveled distance (1A) and escape latency (1B) for the control, sham, Aβ1-42 treated group, crocin pretreated and crocin post-treated in Aβ1-42 injected rats. Aβ1-42 caused a significant increase in traveled distance (*P < *0.001) and escape latency (*P < *0.001) compared to that of the control group. While pretreatment and post-treatment with crocin (30 mg/kg) significantly decreased these parameters compared to those of the Aβ1-42 treated group (*P < *0.001) (Figure 1).

**Figure 2:**
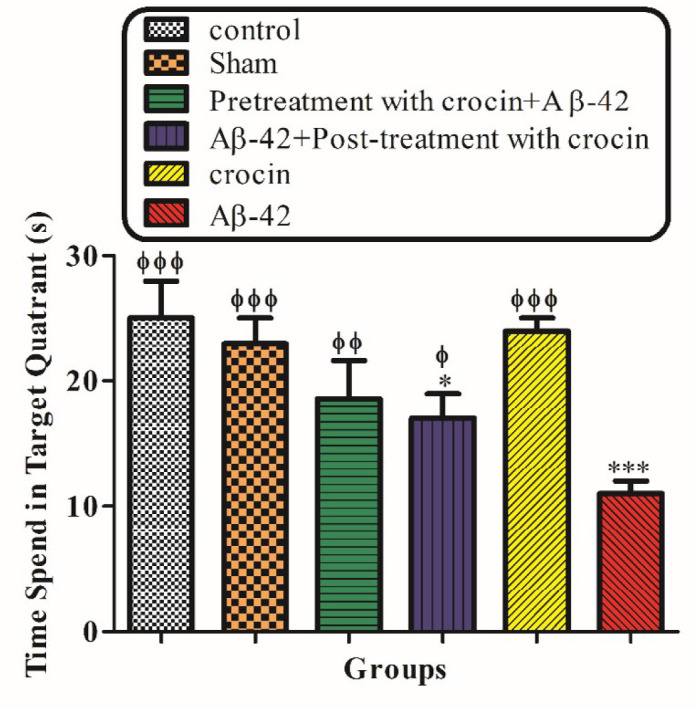
The protective effect of crocin (30 mg/kg) on Aβ1-42 (0.5 μL per side, IH) injected animals on the time that spent in the target quadrant in the probe trial test of the MWM. ^*^P < 0.05, ^***^P < 0.001 compared to the control group. ^ϕ^*P < *0.05, ^ϕϕ^*P < *0.01, ^ϕϕϕ^*P < *0.001 compare to the Aβ1-42 injected animals. The results for each group are presented as mean ± SD for 7 animals in each group. Statistical significance between the groups was determined by one-way analysis of variance (ANOVA) using a Bonferroni post hoc multiple comparison test

Figure 2 demonstrated the result of the probe trial test. The time that rats spend in the target quadrant was significantly decreased in the Aβ1-42 injected group (*P < *0.001) compare to control, but post-treatment (*P < *0.01) and pretreatment (*P < *0.001) with crocin (30 mg/kg) significantly increase this parameter compare to the Aβ1-42 injected group (Figure 2).

*Hippocampal mitochondrial function*

*ROS formation*

**Figure 3:**
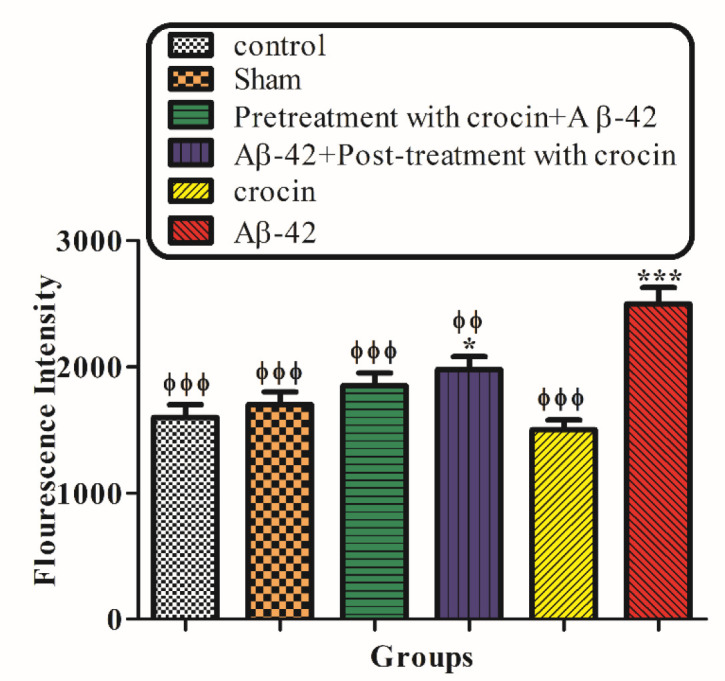
The protective effect of crocin (30 mg/kg) on Aβ1-42 (0.5 μL per side, IH) injected animals on the hippocampal mitochondrial ROS formation. ^*^P < 0.05, ^***^P < 0.001 compared to the control group. ^ϕϕ^*P < *0.01, ^ϕϕϕ^*P < *0.001 compare to the Aβ1-42 injected animals

The result of the ROS generation test demonstrated that the injection of Aβ1-42 (IH) significantly (*P < *0.001) increased the amount of ROS in hippocampal mitochondria compared to the control group. On the other hand, post-treatment (*P < *0.01) and pretreatment (*P < *0.001) with crocin (30 mg/kg) significantly decreased the amount of ROS formation compare to the Aβ1-42 injected group (Figure 3). 

*Mitochondrial membrane potential*

**Figure 4:**
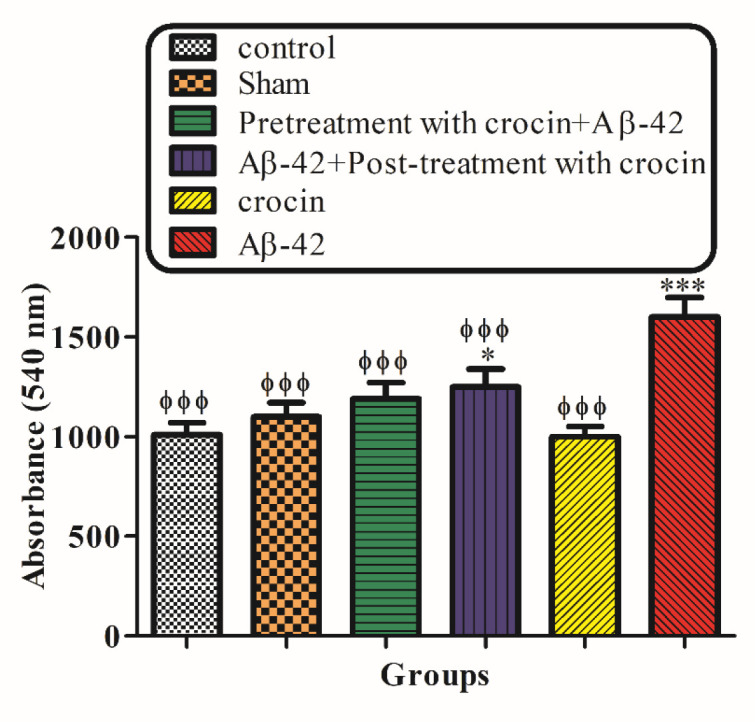
The protective effect of crocin (30 mg/kg) against Aβ1-42 (0.5 μL per side, IH) induced mitochondrial membrane potential decrease. Isolated mitochondria (0.5 mg/mL) were incubated for 1 h. ^*^P < 0.05, ^***^P < 0.001 compared to the control group. ^ϕϕϕ^*P < *0.001 compare to the Aβ1-42 injected animals. The results for each group are presented as mean ± SD for 7 animals in each group. Statistical significance between the groups was determined by one-way analysis of variance (ANOVA) using a Bonferroni post hoc multiple comparison test

Figure 4 showed the effect of Aβ1-42 on mitochondrial membrane potential (MMP) measured using Rh 123 staining test. The MMP significantly decreased in the hippocampal mitochondrial of Aβ1-42 injected rats (*P < *0.001) compare to the control group. The post-treatment and pretreatment with crocin (30 mg/kg) significantly (*P < *0.001) inhibited the collapse of MMP induced by Aβ1-42 (Figure 4).

*Mitochondrial Swelling*

**Figure 5:**
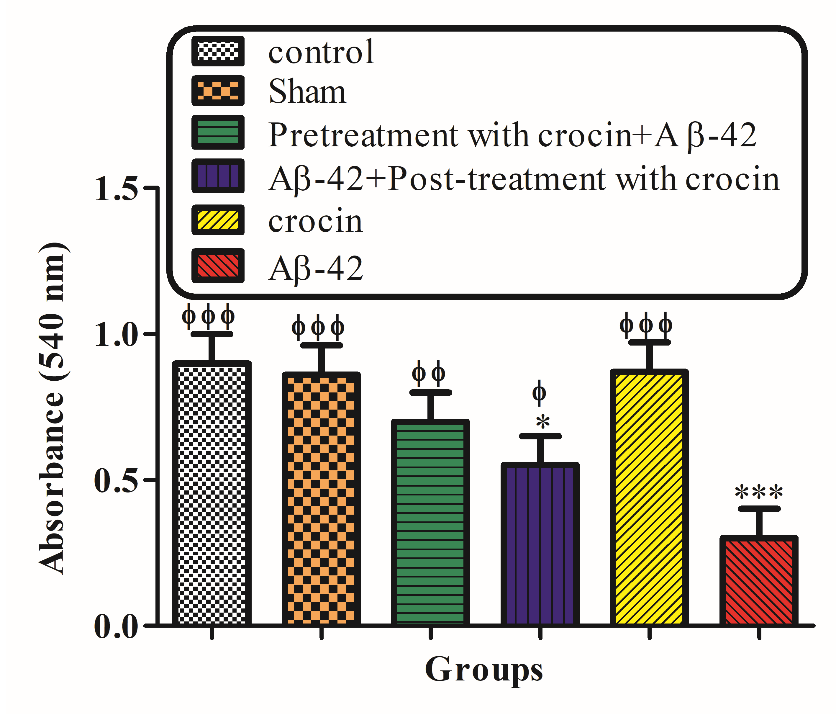
The protective effect of crocin against Aβ1-42 (0.5 μ: per side, IH) induced mitochondrial swelling. ^*^P < 0.05, ^**^P < 0.01, ^***^P < 0.001 compare to the control group. ^ϕ^*P < *0.05, ^ϕϕ^*P < *0.01, ^ϕϕϕ^*P < *0.001 compared to the Aβ1-42 injected animals. The results for each group are presented as mean ±SD for 7 animals in each group. Statistical significance between the groups was determined by one-way analysis of variance (ANOVA) using a Bonferroni post hoc multiple comparison test

Mitochondrial swelling, an indicator of mitochondrial membrane permeability, was monitored with absorbance changes at 540 nm. A decrease in absorbance indicates an increase in mitochondrial swelling. The result in Figure 5 demonstrated that mitochondrial swelling increased according to the injection of A-42 (IH) (*P < *0.05) significantly compared to the control group. Pretreatment (*P < *0.001) and post-treatment (*P < *0.05) with crocin significantly decreased the swelling caused by Aβ1-42 (Figure 5).

*The Cytochrome c Oxidase Activity and Outer Mitochondrial Membrane Damage*

**Figure 6:**
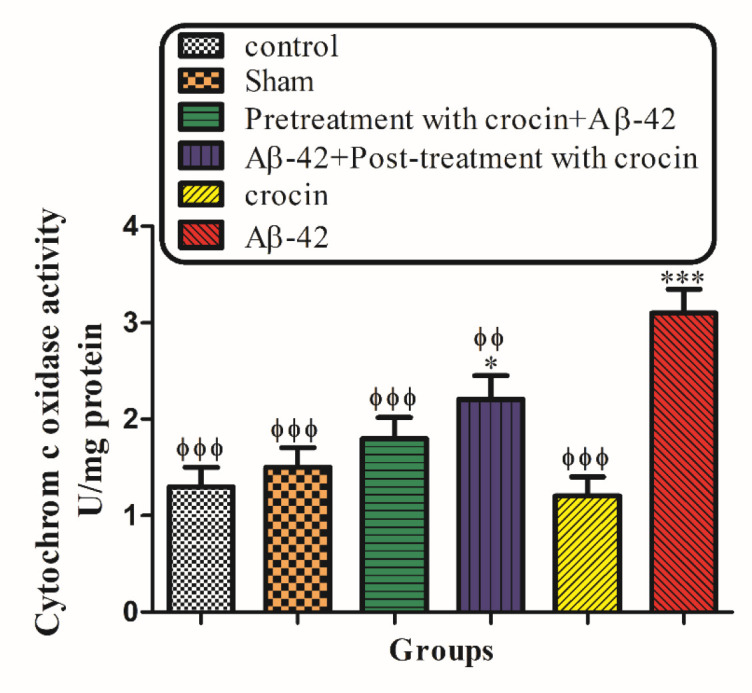
The protective effect of crocin against Aβ1-42 induced hippocampal mitochondrial outer membrane damages. ^*^P < 0.05, ^***^P < 0.001 compared to the control group. ^ϕ^*P < *0.05, ^ϕϕ^*P < *0.01, ^ϕϕϕ^*P < *0.001 compared to the Aβ1-42 injected animals. The results for each group are presented as mean ± SD for 7 animals in each group. Statistical significance between the groups was determined by one-way analysis of variance (ANOVA) using a Bonferroni post hoc multiple comparison test

As shown in Figure 6, cytochrome c oxidase activity was measured in the presence/absence of detergent n-dodecyl b--maltoside. This ratio represents the percentage of mitochondrial outer membrane damage. Our results demonstrated that Aβ1-42 induced mitochondrial outer membrane damages significantly (*P < *0.001) increased compared to the control group. On the other hand, pretreatment (*P < *0.001) and post-treatment (*P < *0.01) with crocin significantly decreased the hippocampal mitochondrial outer membrane damages compared to the Aβ1-42 injected group (Figure 6).

*Lipid Peroxidation*

**Figure 7:**
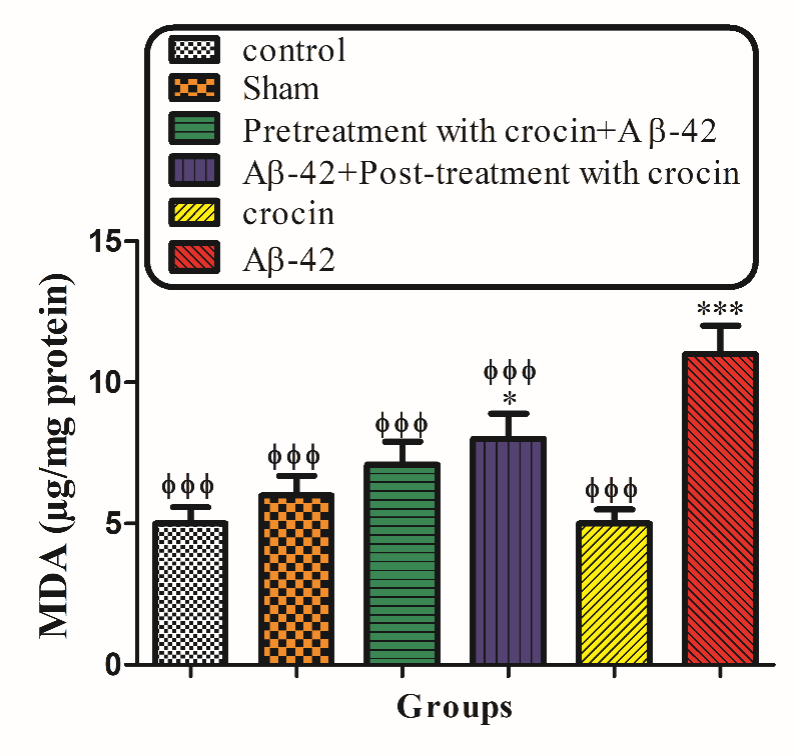
The protective effect of crocin against Aβ1-42 induced lipid peroxidation. ^*^P < 0.05, ^***^P < 0.001 compared to the control group. ^ϕϕ^*P < *0.01, ^ϕϕϕ^*P < *0.001 compare to the Aβ1-42 injected animals. The results for each group are presented as mean ± SD for 7 animals in each group. Statistical significance between the groups was determined by one-way analysis of variance (ANOVA) using a Bonferroni post hoc multiple comparison test

The intra-hippocampal injection of Aβ1-42 to the rats significantly (*P < *0.001) increased MDA formation compared to their corresponding control group. However, pretreatment and post-treatment with crocin at a concentration of 30 mg/kg significantly (*P < *0.001) reduced Aβ1-42 induced lipid peroxidation (Figure 7).

*GSH contents*

**Figure 8:**
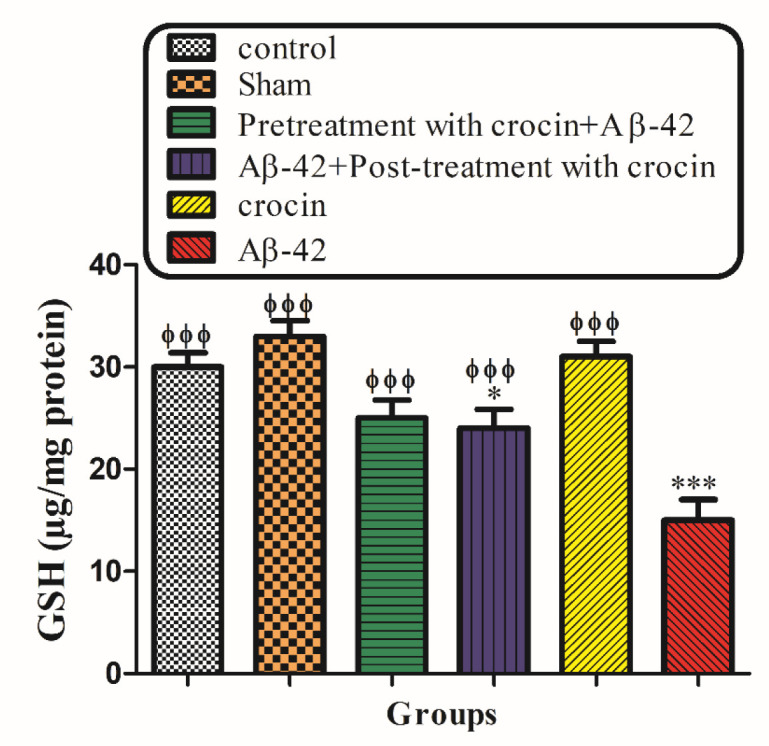
The protective effect of crocin against Aβ1-42 induced GSH depletion. ^*^P < 0.05, ^***^P < 0.001 compared to the control group. ^ϕϕϕ^*P < *0.001 compare to the Aβ1-42 injected animals. The results for each group are presented as mean±SD for 7 animals in each group. Statistical significance between the groups was determined by one-way analysis of variance (ANOVA) using a Bonferroni post hoc multiple comparison test

Mitochondrial GSH, a very important antioxidant defense against ROS formation, was measured spectrophotometrically using DTNB as an indicator in isolated mitochondria after 1 h of incubation. GSH levels in the Aβ1-42 injected group significantly (*P < *0.001) decreased compared to control mitochondria. On the other hand, post-treatment and pretreatment with crocin significantly (*P < *0.001) increased the Aβ1-42 induced GSH depletion compared to the Aβ1-42 group (Figure 8).

*Cytochrome c release*

**Figure 9:**
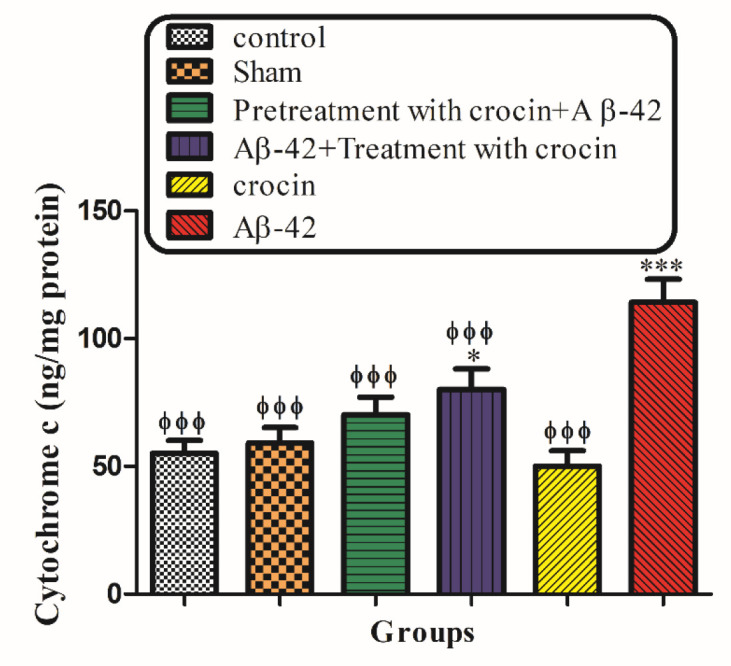
The protective effect of crocin (30 mg/kg) on Aβ1-42 induced cytochrome c release. ^*^P < 0.05, ^***^P < 0.001 compared to the control group. ^ϕϕϕ^*P < *0.001 compare to the Aβ1-42 injected animals. The results for each group are presented as mean ± SD for 7 animals in each group. Statistical significance between the groups was determined by one-way analysis of variance (ANOVA) using a Bonferroni post hoc multiple comparison test

As shown in Figure 9, the collapse of the mitochondrial membrane potential and disruption of mitochondrial outer membrane integrity significantly (*P < *0.001) occurred in the hippocampal mitochondria of the Aβ1-42 injected group compared to the control group. The post-treatment and pretreatment with crocin at a concentration of 30 mg/kg significantly (*P < *0.001) inhibited cytochrome c release (Figure 9).

## Discussion

The antioxidant and protective effects of crocin have been studied extensively before (39). However, no data exist regarding the interaction of crocin with the mitochondrial targeting of Aβ1-42 to prevent Alzheimer’s disease. Also, there is no data that compare the protective effect of crocin (30 mg/kg, IP) while pretreated or post-treated against memory deficiency induced by beta amyloid-42 (0.1 µg/µL, 0.5 μL per side, IH). In the current study, the antioxidant activity of crocin was evaluated in an *in-vivo* experimental model. Furthermore, for evaluating the mechanical and mitochondrial targeting aspects, mitochondrial studies were performed on mitochondria isolated from the rat hippocampus (40).

The results of this study showed that Aβ1-42 administration markedly induced learning and memory impairments. Crocin with its antioxidant and neuroprotective effects, reduced the symptoms of memory deficiency. Previous studies demonstrated that crocin antioxidant properties are comparable to alpha-tocopherol (41). It has also been shown that crocin is effective for memory impairment caused by global cerebral ischemia/reperfusion (RR) (46). Many research projects are devoted to the neuroprotective properties of crocin and its effectiveness for the treatment of neurodegenerative diseases such as aging, Alzheimer’s, and Parkinson’s disease (43). An *in-vitro* study by Papandreou *et al.* showed that crocin has a significant inhibitory effect on the formation of Aβ1-42 (42). Some cellular studies demonstrated that crocin also could prevent neuronal cell death (24). These results suggest that crocin has an effective role in the treatment or pretreatment of AD. Although many studies were investigated the effect of crocin on AD, there is no study to investigate Alzheimer’s mitochondrial complications to the best of our knowledge.

As shown in Figures 1 and 2, our behavioral results imply that crocin effectively improves memory deficiency as a result of Aβ1-42 injection. An interesting finding in the current study is the pretreatment effect of crocin before Aβ1-42 administration which reduced the Alzheimer’s disease symptoms even better than what was seen in the post-treatment of the compound. Crocin has a reducing effect on inflammation (44,45) and ischemia (46). Many studies have illustrated the antiradical scavenging efficacy of crocin (47). It seems that neuroprotective, anti-aging, anti-inflammatory, and antitumor activities of crocin arise from its antioxidant effects (48-50). Soeda *et al.* Showed that the efficacy of crocin at the inhibition of oxidative stress-induced cell death is due to a GSH-dependent mechanism (51). Therefore, it seems that these antioxidant and neuroprotective effects of crocin are better seen when the cells are still alive or in the early damage steps.

Previous studies showed that mitochondria might have a pathogenic role in human disease. This typically affected organs with greater energy requirements, such as the central nervous system. (52). Faizi *et al.* confirmed that the generation of reactive oxygen species following Aβ1-42 exposure was increased in isolated rat hippocampal mitochondria (53). In our study, when Aβ1-42 was injected into the hippocampus of rats, ROS levels were increased, but this increased amount of ROS was reduced by pretreatment and post-treatment with crocin. This study showed that crocin as an antioxidant can prevent the harmful effects of Aβ1-42 on hippocampal mitochondria. As clear, there is a relationship between molecular structure and the antioxidant effect of crocin. It is accepted that the mechanistic pathways for scavenging free radicals by crocin are similar to those of the most known non-polar carotenoids (54). Previous manuscripts confirmed a direct relationship between the antioxidant effect of the compounds and their double bind that exist in their molecular structure. Crocin has more double bonds than α-tocopherol in the molecule. Therefore, we can conclude that crocin may have a more antioxidant effect than α-tocopherol. (51).

Mitochondria are substantial sources of reactive oxygen species in the cell. Because about 1 - 2% of the total oxygen consumed by mitochondria is converted to superoxide anion in the mitochondrial respiratory chain, followed by its rapid conversion to H_2_O_2_ through mitochondrial superoxide dismutase (SOD). After the injection of Aβ1-42, the increase in H_2_O_2_ formation occurred in isolated hippocampal mitochondria, suggesting the potential role of mitochondrial H_2_O_2_ in neurotoxicity (44). Our results demonstrated that crocin reacts with superoxide anions and H_2_O_2 _in mitochondria and makes them ineffective. Therefore, crocin can prevent neuronal damage caused by Aβ1-42 through oxidative stress. 

The administration of Aβ1-42 significantly (*P < *0.001) increased lipid peroxidation in isolated hippocampal mitochondria. This phenomenon indicates that one of the early targets of oxygen free radicals is mitochondrial lipids because of their high content of unsaturated fatty acids. Besides, the oxidation of the lipid membrane disrupts the mitochondrial membrane and induces the collapse of MMP. Our results demonstrated that crocin with its antioxidant activity, prevented mitochondrial membrane lipid peroxidation in brain neurons caused by Aβ1-42. In previous studies, the effect of crocin on preventing lipid peroxidation and maintaining the lipid membrane of the cells was proven (55).

In addition to the enhanced ROS formation, Aβ1-42 also can decrease the level of cellular glutathione (GSH plays a critical role in maintaining cellular redox homeostasis) (56). The oxidation of mitochondrial GSH has a greater toxic effect on cell viability than cytosolic GSH oxidation (57). This is because GSH is an antioxidant and an essential factor for maintaining thiol groups of mitochondrial proteins in the reduced state (58). Our result demonstrated that crocin significantly increased the amount of GSH. The effects of crocin on GSH may be attributed to its direct antioxidative effect or its intensifying role in the biosynthesis of GSH (59). Although pretreatment with crocin seem to affect much better than post-treatment with it; there is no significant difference between the two groups in increasing the amount of intracellular GSH.

In our study, the opening of the MPT pores by Aβ1-42 caused mitochondrial swelling, which is after cytochrome c release. This finding is exactly according to previous studies showing that Aβ1-42 caused a significant collapse of MMP in treated cells. Besides, the addition of crocin significantly inhibited the Aβ1-42 induced MMP collapse and mitochondrial swelling, suggesting that oxidative stress is directly involved in MMP collapse due to MPT pore opening. 

Our result indicated that Aβ1-42 significantly increased the mitochondrial outer membrane damage. Aβ1-42 induced lipid oxidation in liver mitochondria could promote mitochondrial membrane damage and, finally, the loss of mitochondrial outer membrane integrity. Crocin prevented internal membrane damage caused by Aβ1-42 by the inhibition of lipid peroxidation.

It is proven that both the corruption of mitochondrial membrane potential and cytochrome c release are important indicators of cellular apoptosis and key endpoints for determining the mitochondrial dysfunction (60). Our results showed that Aβ1-42 caused the significant expulsion of cytochrome c from mitochondria. Moreover, crocin post-treatment and pretreatment blocked the Aβ1-42 induced release of cytochrome c from the isolated hippocampal mitochondria, which supports the hypothesis that the apoptosis induced by Aβ1-42 is due to oxidative stress and depends on the opening of the MPT pores.

Although crocin could reduce the behavioral signs and hippocampal mitochondrial toxic parameters induced by Aβ1-42 administration, the results in the plot showed that in almost all tests, pretreatment with crocin before Aβ1-42 injection (IH) could reduce these parameters better than treatment with crocin after Aβ1-42 injection. However, there was not a significant difference between these two groups. 

The difference between the crocin-based post-treatment and pretreatment effects mentioned above is due to the effect of crocin on increasing intracellular antioxidant capacity. With increasing intracellular glutathione levels, Crocin prepares the cells for interaction with an oxidative stress-causing agent. On the other hand, crocin treatment also protects cells well because of its antioxidant mechanisms and reduces neurotoxicity and slowing the progression of the disease. This study suggests that the earlier protection of the neurons could lead to a reduction in the neuron’s loss and the incidence of Alzheimer’s disease.

The results for each group are presented as mean ± SD for 7 animals in each group. Statistical significance between the groups was determined by one-way analysis of variance (ANOVA) using a Bonferroni post hoc multiple comparison test.

## Conclusion

To sum up, based on the above findings, the mitochondrial mechanisms associated with Aβ1-42 induced Alzheimer’s are the increasing of ROS formation, lipid peroxidation, GSH oxidation, mitochondrial permeability transition pore opening, mitochondrial, swelling and cytochrome c release. However, Aβ1-42 induced neurotoxicity can be prevented by crocin through these pathways. We can conclude that crocin is a potent antioxidant that protects the neuronal mitochondria against the development of Alzheimer’s disease. Therefore, it is recommended to use crocin to reduce the risk of Alzheimer’s disease at an advanced age. 
